# Visualization and workload with implicit fNIRS-based BCI: toward a real-time memory prosthesis with fNIRS

**DOI:** 10.3389/fnrgo.2025.1550629

**Published:** 2025-05-06

**Authors:** Matthew Russell, Samuel Hincks, Liang Wang, Amin Babar, Zaiyi Chen, Zachary White, Robert J. K. Jacob

**Affiliations:** Computer Science, Tufts University, Medford, MA, United States

**Keywords:** HCI, BCI, fNIRS, implicit BCI, memory prosthesis, default mode network, task positive network

## Abstract

Functional Near-Infrared Spectroscopy (fNIRS) has proven in recent time to be a reliable workload-detection tool, usable in real-time implicit Brain-Computer Interfaces. But what can be done in terms of application of neural measurements of the prefrontal cortex beyond mental workload? We trained and tested a first prototype example of a memory prosthesis leveraging a real-time implicit fNIRS-based BCI interface intended to present information appropriate to a user's current brain state from moment to moment. Our prototype implementation used data from two tasks designed to interface with different brain networks: a creative visualization task intended to engage the Default Mode Network (DMN), and a complex knowledge-worker task to engage the Dorsolateral Prefrontal Cortex (DLPFC). Performance of 71% from leave-one-out cross-validation across participants indicates that such tasks are differentiable, which is promising for the development of future applied fNIRS-based BCI systems. Further, analyses within lateral and medial left prefrontal areas indicates promising approaches for future classification.

## 1 Introduction

This work specifically intends to push the boundaries of implicit BCI with prefrontal cortex measurements using fNIRS. Implicit BCI centers on interactions where the interface recognizes the current brain state of the user and adapts accordingly, without the human user's purposeful intent (George, 2010; Zander and Krol, 2014; Treacy Solovey et al., 2015). These BCIs do not require conscious thought to direct the interface; they are like a helpful assistant. Consider a screen that self-adjusts brightness levels depending on ambient light—implicit BCI systems do just the same, but with brain state as the driving force of the interface. fNIRS is a noninvasive and relatively portable tool (Ferrari and Quaresima, 2012) which measures changes in oxygenated and deoxygenated hemoglobin in the blood (Izzetoglu et al., 2005).

A considerable number of fNIRS based implicit BCI studies have been done using prefrontal cortex activation to approximate mental workload. Some have been real-time tasks (Girouard, 2013; Afergan et al., 2014a, 2015, 2014b; Hirshfield et al., 2011, 2009b,a), while others are offline studies attempting to distinguish brain states (Power and Chau, 2010; Strait, 2014). Most studies infer mental workload by first training a model based on an N-Back task which later is used to modulate the difficulty of a separate task in real time (Afergan et al., 2014a; Shibata et al., 2019; Strait, 2014; Yuksel et al., 2016; Afergan et al., 2015, 2014b).

Our study departs from the trend and instead follows the paradigm established by Hincks (2019) in that, rather than infer levels of workload, we instead developed specifically intended to engage with the prefrontal cortex in different ways: to infer Default Mode Network (DMN) activity through creativity (Beaty et al., 2014), and Dorsolateral Prefrontal Cortex (DLPFC) activity through working memory (Barbey et al., 2013). Demonstration of an interface leveraging brain-network activation orientation for classification can open the door for future useful adaptations based on the balance of brain network activation patterns Hincks (2019).

In terms of application, our prototype system is motivated to step toward the notion of a general brain-based “assistant” that helps its user recall items by indexing them according to their mental state and presenting relevant information automatically, rather like the “memory prosthesis” first introduced in the work of Rhodes (1997) and Lieberman (1995), but with passively measured brain state as the storage and retrieval tag. We can envision in the future a brain-based interface which is able to recognize and adapt fluidly to a user's brain state—such a system, as a memory prosthesis, would both be able to store brain states associated with important information, and to provide such information when the user requires it.

Such an associative memory assistant could be useful in a variety of common knowledge worker research tasks. Examples range from examining and organizing text a body of legal documents for a lawyer, to surveying papers for an academic survey or policy analysis, to businesses analysis for acquisition or valuation. In a conventional filing system, the user could store such items in a bookmarked list as they are reviewed and then retrieve them from it later.

Furthermore, bookmark creation will be able to be automatically generated based on analysis of the brain signal. Bookmarks will then be ordered by how well each one matches the current brain state. Thus, whenever a user sees the list, they will first see those items that they entered while in the same brain state as they are in currently. The rationale is that these might be the most relevant items for the user at the current moment. The benefit is that the system would display them automatically and continuously, without any user effort, without scrolling through a variety of previously stored bookmarks nor having to enter tags explicitly. The filing system index is simply the user's passively measured brain state. Of course, in a more practical system, the filing system would permit other indexes as well.

Beyond the basic low-level interaction speed advantage of having the top bookmarks preselected effortlessly, Gray and Boehm-Davis (2001) provide experimental evidence of a direct impact of such rapid, low-level, lightweight interaction on a user's higher level strategy and behavior; it can produce changes well beyond the actual speedup of the improved low-level interaction. They observe that a slight change in an interface can shift subjects from a trial-and-error problem solving approach to a plan-based one. Instead of displaying content near the user's current state, some work suggests that it might be better to display content semantically far removed, in a creative ideation task (Chan et al., 2017). Our system could directly support either approach.

As described below, our prototype is designed to take a step toward this higher level vision while initially reducing some of its complexities. We assume the user is alternating between only two specific tasks; and for now, we use task-classification as a proxy for bookmarking process. Our prototype runs in real-time to demonstrate the general feasibility of our memory assistant design. We defined two tasks that could be done by an experimental subject without particular domain expertise and that were intended to elicit two different measurable brain states, and we investigated our ability to distinguish them passively and in real time.

## 2 Materials and methods

### 2.1 Task design

After iterative pilot testing, we chose to work with a broad task that an at-home user might experience: designing a room in their home or apartment. Participants were given three rooms to design: a Living Room, Bedroom, and Dining Room. We subdivided the broad task of room design into two phases—the *inspiration phase* (Task A), in which the the goal was to observe images of a room similar to the one they were being asked to design—and the *furniture selection* phase (Task B), wherein they chose furniture for their room. We chose these two tasks precisely in an attempt to interface with the prefrontal cortex in different ways, and for their similarity with real-world tasks a user might perform in their home. Both of our experimental tasks are open-ended by design, and require a complex set of thought processes that are unscripted and non-trivial.

#### 2.1.1 Visualization phase

During the *Visualization phase*, participants were provided a sidebar of small image links of the room they were assigned to design. We gathered stock photos of example Living Rooms, Bedrooms, and Dining Rooms. During this phase, the participant's task was explicitly limited to clicking on the sidebar image links, observing the larger images that would appear as a result of clicking on the links, and considering what they would like for their own room. Although visual prompts were provided, participants were instructed to use these images as inspiration for their own internal thoughts of what they would like to create; that is, this task was specifically designed to engage spontaneous cognition and internally directed thought (Buckner et al., 2008; Bartoli et al., 2024), and therefore is a proxy for applied DMN-based tasks.

#### 2.1.2 Furniture selection phase

During this phase, participants were tasked with browsing items from the Ikea website. They were further responsible for keeping track of items they would like to purchase in a Google Sheets spreadsheet. During this task, participants were assigned a budget of $750 USD per room, which they were trying to maximize use of. Similar to Task A, we also provided a sidebar with photo links, but these were of Ikea furniture items that linked to the corresponding items on the website (instead of to an image viewer program)—each of these items, we mentioned to participants, were to be discounted by 50%. They were to calculate prices using a calculator or spreadsheet calculator if desired, and keep track of the totals in the spreadsheet. Participants were asked to choose at least five items during each phase. Through the combination of multitasking, numeric calculation, and high time pressure, this task was specifically designed to engage with the DLPFC (Modi et al., 2020; Mahesan et al., 2023).

### 2.2 Equipment

**Figure 1:**
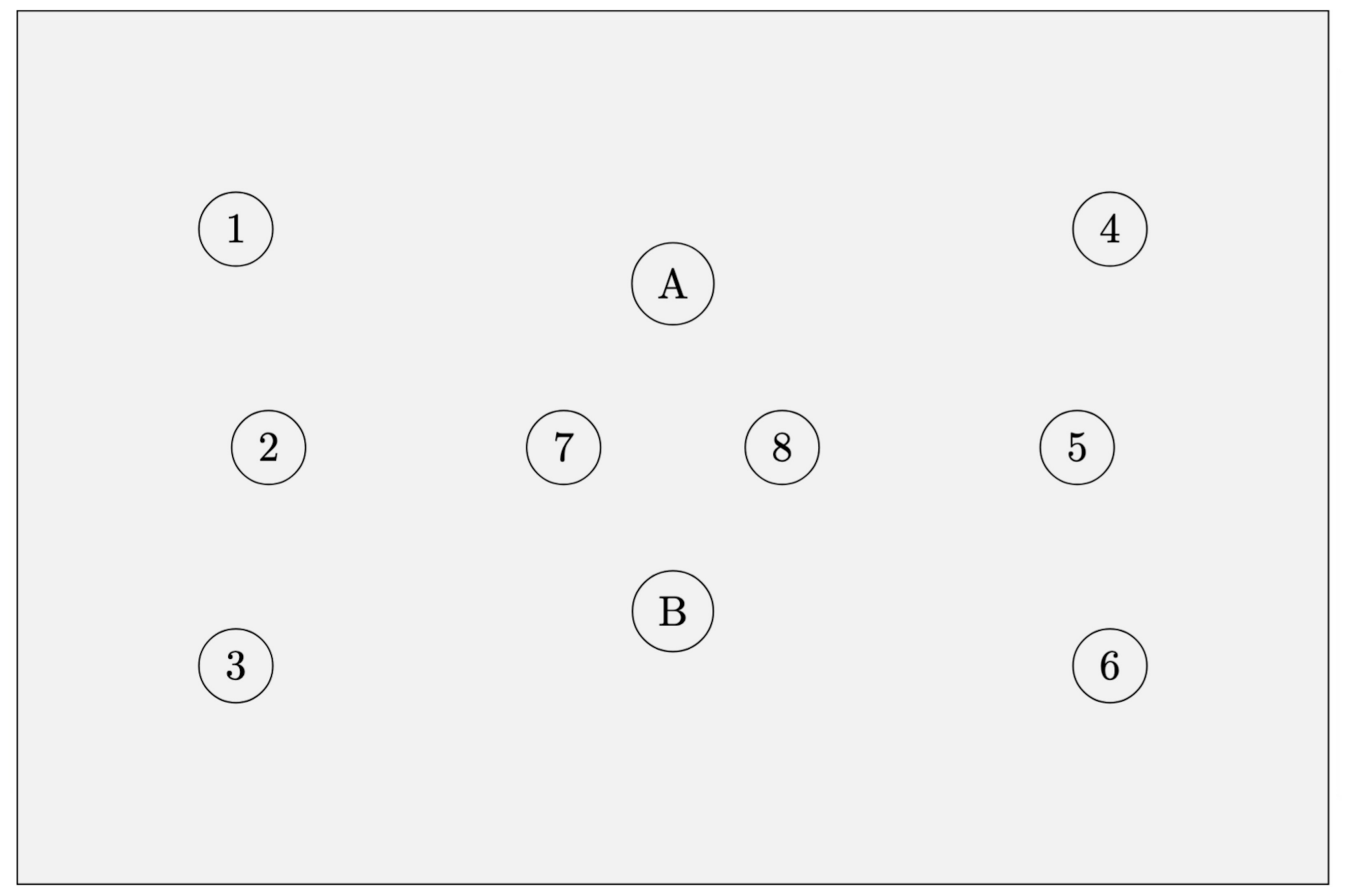
fNIRS probe geometry. Eight source locations with two detector locations (A, B). Each source location contains two light sources, one at 830 nm and the other 690 nm. Source locations 7 and 8 are used only for short source-detector pair adaptive filtering to remove extracerebral data. Short sources are 1.5 cm from each detector; for each detector, the nearest 4 source locations (outside of the short sources) are each 3 cm from the detector, and the furthest two source locations are 3.61 cm from the detector. Due to technical issues data could only be collected from the B detector for this study.

**Figure 2:**
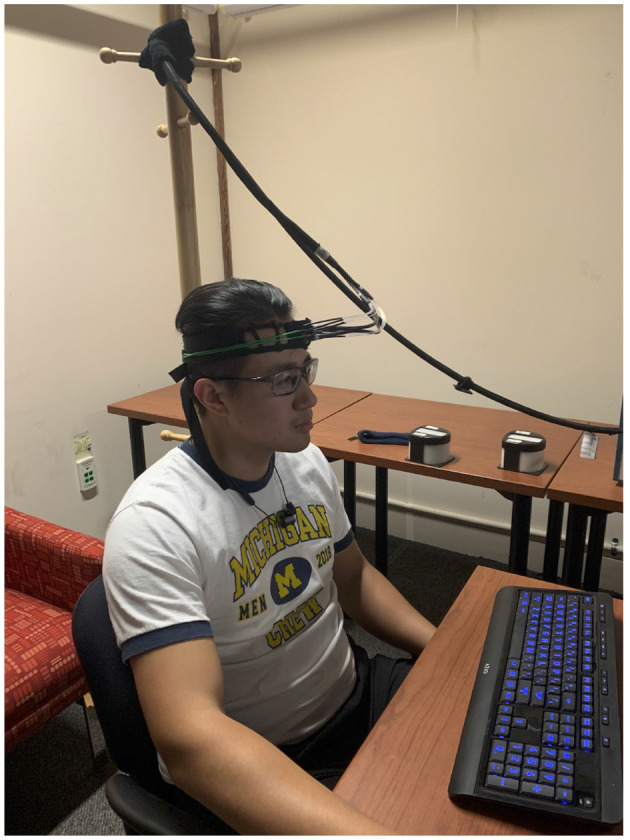
fNIRS headband on a participant (photo taken with consent). The probe pad is placed over the left eyebrow at approximately the Brodmann 10 region.

We used a Multichannel ISS Imagent fNIRS device (Champaign, IL) for our data acquisition. We used a single probe pad with two detectors and eight light source positions (see Figure 1 for details). Two source positions were close sources (1.5 cm) used for near source-detector pair adaptive filtering (Zhang, 2007). The probe pad was positioned over the left eyebrow at approximately the left prefrontal cortex Brodmann 10 region (Figure 2). Due to the probe pad geometry, one set of three light source positions was over a relatively lateral aspect of Brodmann 10, and the other set of the light sources were over a relatively medial aspect. However, the detector itself was in the center of source positions. Outside of the near sources, the closest 4 source positions were each 3cm from the detectors, and the furthest two source positions were 3.61 cm from the detectors. Each light source position had two sources which emit infrared light at one of two near-infrared wavelengths (830 nm and 690 nm) (Kocsis et al., 2006). Raw Alternating Current (AC), Direct Current (DC), and Phase values were converted via the Modified Beer-Lambert Law to Delta Oxygenated and Deoxygenated Hemoglobin values (HbO and HbR) (Kocsis et al., 2006). Data was acquired at approximately 5.8 Hz; real-time data were bandpass filtered from 0.1 to 0.4 Hz (Kirilina et al., 2012), which enables us to isolate the physiologically relevant hemodynamic response signals from cardiac and Mayer waves (Naseer, 2015; Seghouane and Ferrari, 2019). Unfortunately, we encountered data acquisition issues in one of the two detectors, therefore only a single detector was used for this study. Although the single detector was able to capture prefrontal data from relatively lateral and medial aspects of the prefrontal cortex, the limitation of the single detector reduced our ability to capture the vertical spatial distribution of hemodynamic responses over the prefrontal area. The second detector would have allowed for more comprehensive mapping of activation patterns and potentially improved classification accuracy by providing both redundancy in some aspects of measurement and broader spatial coverage in others.

### 2.3 Participants

After initially prototyping our study with 6 participants (4 male, aged 18 to 23 years, mean age 20.1, sd 1.2), we recruited 8 participants for the study (6 male, aged 18–27 years, mean age of 20.6, sd 2.8). All participants reported being right-handed. None reported having had either traumatic head injury or learning or reading disability. All reported normal/corrected-to-normal vision.

### 2.4 Experiment design

**Figure 3:**
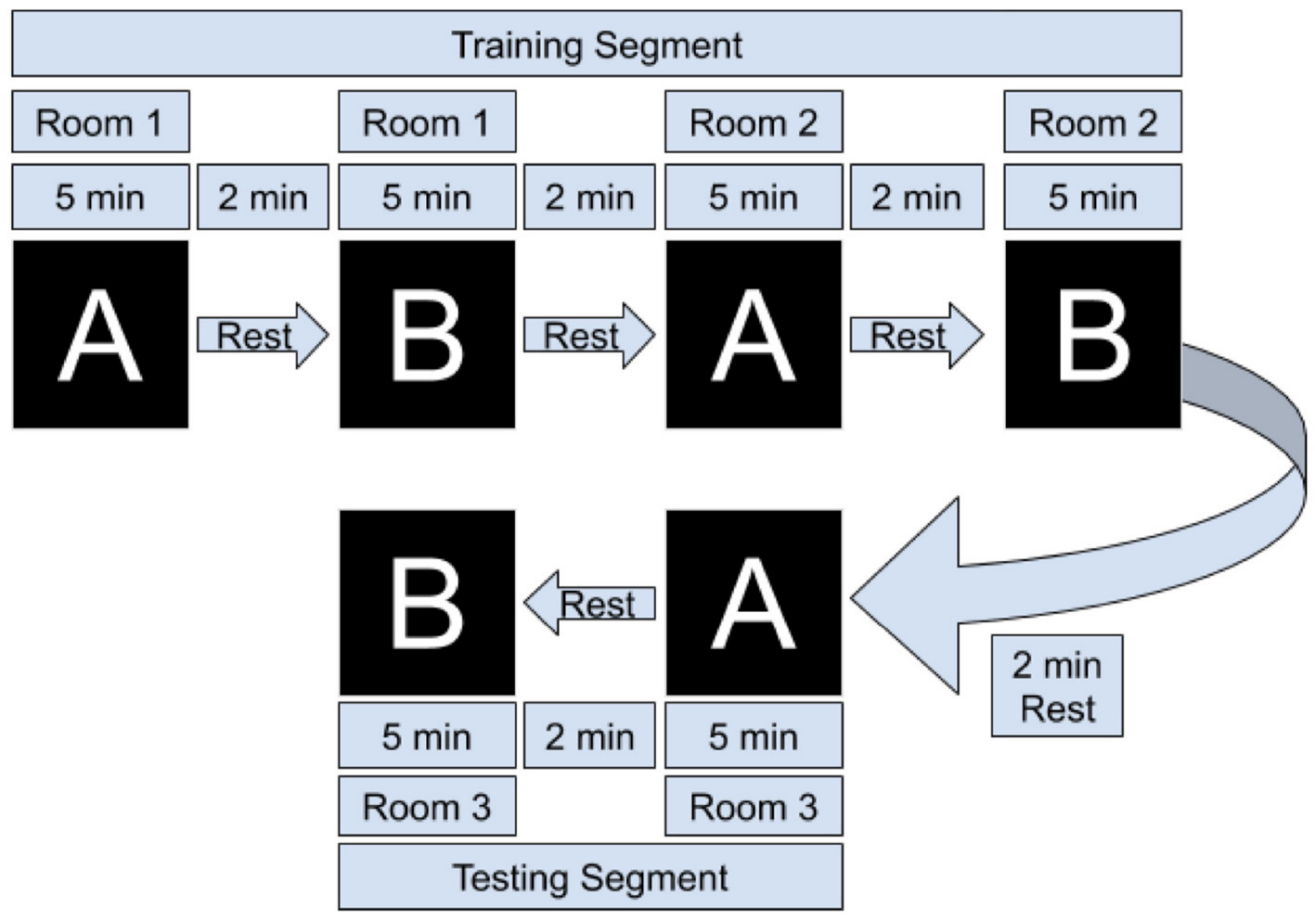
Overview of task flow. Task A refers to the Visualization phase (looking at images), and Task B refers to the furniture selection phase. Each set of A/B consisted of designing a living room, dining room, or bedroom. The adaptive filter coefficients, scaling coefficients, and SVM model are learned/trained after the first set of tasks, and during the final group of two tasks they are tested in real-time by a Python thread that extracts data for classification every 20 s.

Participants sat in a comfortable chair in front of a computer terminal running Red Hat Enterprise Linux 7.7. (Solovey et al., 2009), read and signed a consent form, and filled out a demographic questionnaire. We then explained the tasks to the participants and fitted the fNIRS headband. They then completed two groups of room design tasks, where each group contained one trial of type A, a rest period of 2 min, then one of type B, then a rest period of 2 min. We chose an extended rest period length of 2 min for two reasons: first, to ensure complete dissipation of post-stimulus overshoot of from the BOLD signal (Schroeter et al., 2006), and second, to provide participants with a substantive break time to mentally relax between tasks. See Figure 3 for a visual representation of the task flow. Brain data from the first two groups of tasks were used to train a machine learning model; during the last group of tasks the model was used in real time to classify the user's brain state every 20 s—the user-interface would update to show the links corresponding with the brain state predicted by the model. After the three sets of tasks participants filled out a post-survey questionnaire and were compensated with $25 USD.

### 2.5 Interface details

**Figure 4:**
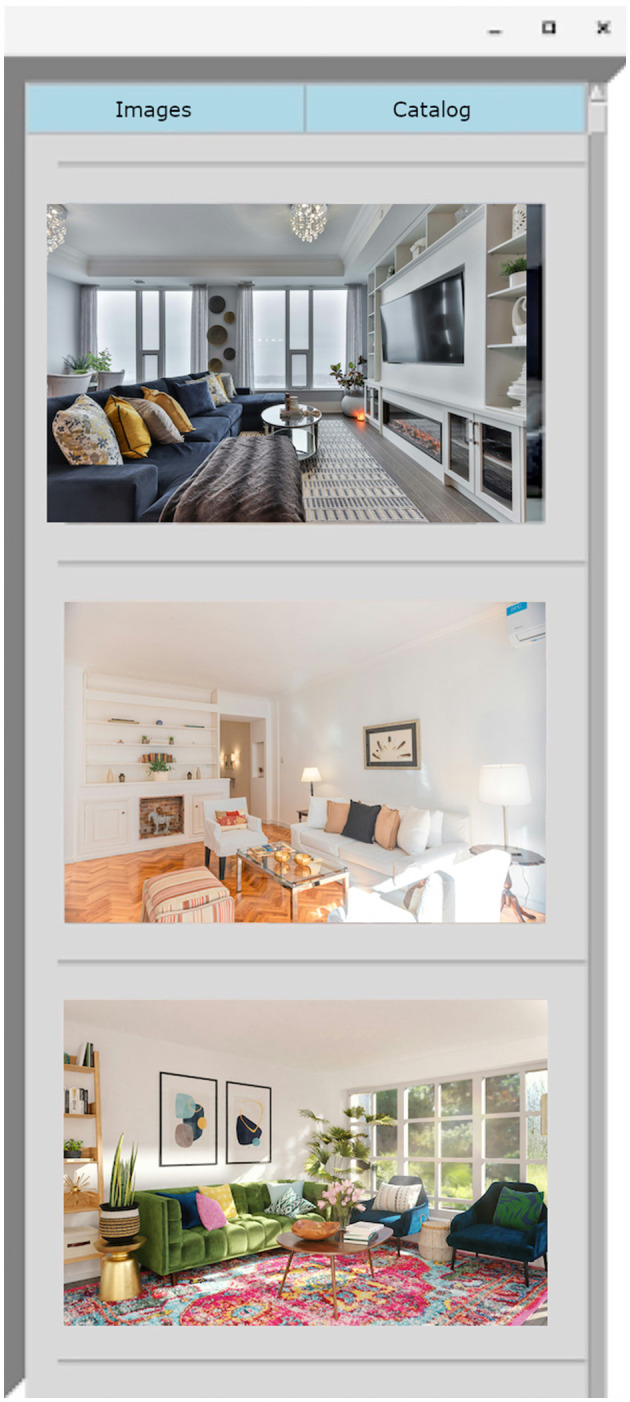
Example sidebar presented in Task A (Visualization phase). Clicking on an image would expand it full-screen.

**Figure 5:**
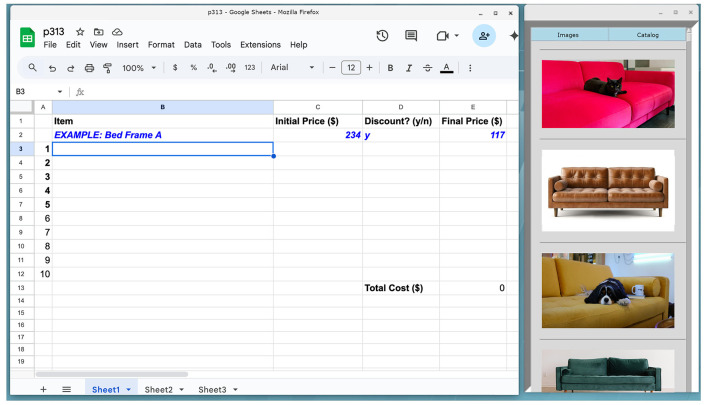
Task B; task tabs on the left, and images on the right would link to the Ikea website; these images were of furniture which would be discounted by 50%.

During the start of each task a window would appear on the user's screen with two buttons—*Images* and *Catalog* (see Figure 4). Users were instructed to press the *Images* button during Task A, and to press the *Catalog* button in Task B. The appropriate images or catalog links would only appear upon pressing the button. During Task A, clicking on the image links would pop out a larger image into an image viewer - users could zoom in to more closely observe the inspiration image if desired. At the beginning of Task B, we opened a Firefox window with two tabs—a Google sheets spreadsheet tab for the participant to keep track of their purchases, and a basic Google web calculator tab (see Figure 5). The sidebar contained images which were links that would open a new tab in the same browser window which would go directly to the Ikea website to an item that was on sale. Participants were given an incentive to select the sidebar links by being instructed to maximize the number of items selected while staying under budget; these sidebar items were discounted at 50% off. During Task B, users were freely allowed to browse the entire Ikea web interface, but they were not allowed to depart from it and the other tabs we had opened. During the last set of trials, machine learning was used to automatically select the sidebar option of interest.

### 2.6 Data filtering and preprocessing

For each trial we used a Recursive Least Squares adaptive filter with our near-channels to remove the effects of neurovascular coupling and movement artifacts (Zhang and Rolfe, 2012; Zhang, 2007). Per Zhang (2007), we filtered HbR and HbO separately. Further, one filter was used for each of the relatively lateral and medial sides of the probe, where each filter associated the near-source with the outer three sources nearest it. Data from the first two groups of trials were used to train coefficients of the RLS filters for HbR and HbO (Zhang, 2007). After filtering the data, we scaled each channel by removing mean and scaling to the unit variance (Pedregosa et al., 2011).

We then divided the data into segments of 100 (17.24 s). This window frame size was chosen in consideration of the balance between temporal resolution and data quality. That is, we use a window which should be able to capture the full dynamics of a single hemodynamic response (Voss, 2016), while maintaining a time-window length within which we can perform useful classification in the context of a real-time interface. We then extracted the max and mean values of each channel for each window (Naseer, 2015). Feature selection and machine learning were implemented via the scikit-learn library (Pedregosa et al., 2011). Specifically, the SelectKBest algorithm (Pedregosa et al., 2011), which leverages F-test results to identify the K most statistically significant features from the original feature set, was used for features selection with the parameter K=10. We then input the data to a Support Vector Machine (SVM) with a Linear Kernel (Solovey et al., 2011), using default parameters including L2 regularization (C = 1.0), squared hinge loss, and 1,000 maximum iterations.

### 2.7 Online classification

We selected the final two trials to perform real-time (“online”) classification. During the final two trials data was extracted every 100 frames (17.24 s), adaptive filter coefficients learned from the training data were used to filter the data, scaling coefficients from the training data were used to scale the data, feature set size was reduced to the same features used in the training set, and data was then classified by the pre-trained SVM. The result of classification led to immediate presentation of the stimulus the participant was attempting to work on: that is, correct classification would show the “correct” links, and incorrect classification required the user to scroll to the top of the list and press the button corresponding to the task they were completing.

### 2.8 Offline classification

We also conducted additional offline analyses to provide more comprehensive results. This analysis allows us to leverage the entire dataset, rather than be limited to a single participant's data for classification.

#### 2.8.1 Leave-one-out cross-validation

We implement participant-level Leave-One-Out Cross-Validation (LOO-CV) to evaluate classification performance. This validation procedure consists of multiple folds, where in each fold we exclude one participant's complete dataset for testing and exclusively use the training cohort's data to determine the preprocessing pipeline parameters, including adaptive filter coefficients and scaling factors. Following preprocessing, model hyperparameters are optimized through an inner cross-validation procedure conducted solely on the training participants. Model results are then generated for the test set. Final classification results are computed by aggregating results across all participant-specific test sets.

#### 2.8.2 Brain-network dependent classification

To investigate the functional specificity of source locations in conjunction with our tasks we conducted analyses within the context of reduced source sets: specifically, in addition to all source data, we also tested removing either the relatively medial or lateral sources. This analysis enables us to explore the utility of relatively lateral and medial aspects of prefrontal cortex activation as associated with the tasks at hand.

#### 2.8.3 Feature selection

For offline classifications we modified our feature selection strategy with a number of improvements. First, we expanded the feature set to include standard deviation, skew, and slope of the linear regression. Second, we tested varying data window sizes, by attempting [50, 100, 150, 200, 250, 300], equivalent to [8.62, 17.24, 25.86, 34.48, 43.1, 51.72] seconds, respectively. Unlike our previous approach, we opted not to utilize the SelectKBest function for feature selection.

#### 2.8.4 Model selection

Following the most successful models used in fNIRS classification from (Naseer et al., 2016), we added K-Nearest Neighbors (KNN) (Bzdok et al., 2018; Naseer et al., 2016), Linear Discriminant Analysis (LDA) (Xanthopoulos et al., 2013), Quadratic Discriminant Analysis (QDA) (Qin, 2018), and Artificial Neural Networks (ANN) (Thanh Hai et al., 2013). Based on further classification in (Huang et al., 2021) we further added Random Forests (RF) (Breiman, 2001). Although deep learning approaches have demonstrated promising results for fNIRS-based BCI (Eastmond et al., 2022), we decided against using these methods due to the substantial time investment required for model preparation and training. However, we recognize the potential value of deep learning methods for future research in this area; to facilitate such work, we will make our dataset publicly available alongside our paper.

We tuned hyperparameters for some models: the KNN classifier was implemented with varying neighborhood sizes (3, 5, 7, and 9); SVM regularization parameter C was evaluated at levels (0.1, 1, and 10); the QDA regularization parameter was tried with levels (0.1, 0.5, 1); ANN was assessed one internal layer of either 10 or 50 nodes, and used fixed maximum iteration count of 5000 to ensure convergence; and the RF classifier was tested with varying numbers of decision trees (10, 50, 100, and 200).

### 2.9 Classification metric

All results reported, for both online and offline results, are macro average F1 scores, which represents the unweighted harmonic mean of precision and recall. Formally, for a K-class problem, macro-averaged metrics are:


(1) $$\begin{array}{l} \text{Macro-Precision}=\frac{1}{K}\overset{K}{\underset{i=1}{\sum }}\frac{TP_{i}}{TP_{i}+FP_{i}} \end{array}$$

(2) $$\begin{array}{l} \text{Macro-Recall}=\frac{1}{K}\overset{K}{\underset{i=1}{\sum }}\frac{TP_{i}}{TP_{i}+FN_{i}} \end{array}$$

(3) $$\begin{array}{l} \text{Macro-F1}=\frac{1}{K}\overset{K}{\underset{i=1}{\sum }}\frac{2\times P_{i}\times R_{i}}{P_{i}+R_{i}} \end{array}$$

where *TP*_*i*_, *FP*_*i*_, and *FN*_*i*_ denote true positives, false positives, and false negatives for class *i*. By giving equal weight to each class, macro-averaging highlights model performance across all classes (Opitz, 2022).

## 3 Results

### 3.1 Real-time results

**Figure 6:**
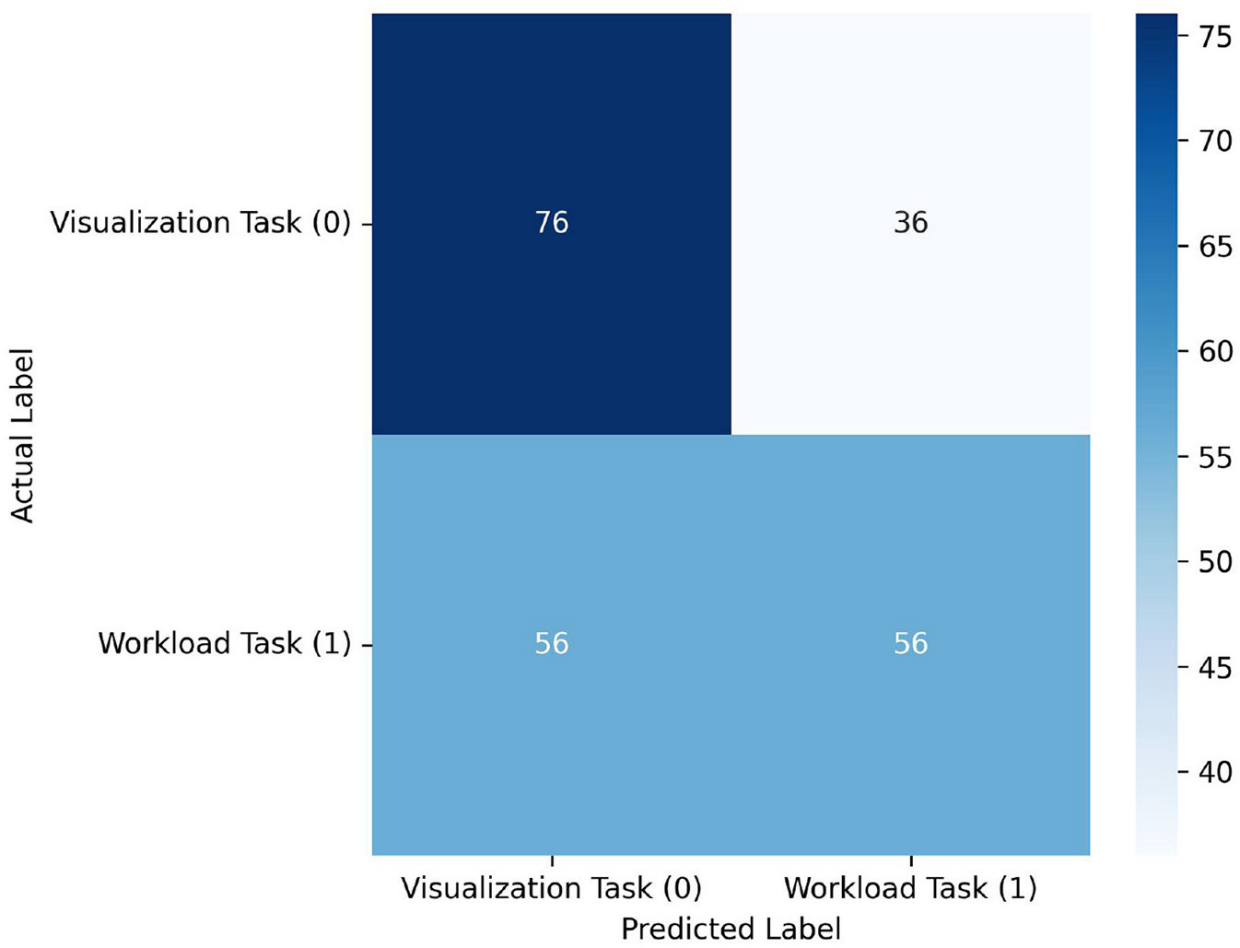
Confusion matrix across all participant predictions in the realtime experiment. Although the model classified a relatively high number of 76 visualization tasks correctly, it struggled to correctly identify workload tasks with only 56 correct classifications. Likewise, it incorrectly classified 56 workload samples as visualization and 36 visualization samples as workload. These results suggest that the model is not capable for usable realtime classification.

See Table 1, Figure 6. Notably, the model's performance varies significantly across different participants. The most successful results were achieved with PID 3, with F1-scores of 0.966 and 0.963 for visualization and workload classes respectively, and a macro average F1-score of 0.964. However, overall performance across participants was inconsistent. Several participants (1, 2, 4, and 6) showed particularly poor results, with F1-scores of 0.000 for one or both classes. The average macro performance across all participants was 0.516 with a substantial standard deviation of 0.282, highlighting the high variability in the model's effectiveness. The confusion matrix in Figure 6 shows that the model overall performed better in classifying Visualization (class 0) compared to Workload (class 1) tasks, although the results table indicates that this pattern was not consistent across all participants. Overall, the realtime results indicate that model used was not sufficiently robust for reliable real-time classification across different users. We believe that the lack of substantial training data is the largest factor in the low overall scores.

**Table 1:** Per-participant results for the online real-time classification.

**Participant**	**Class**	**Precision**	**Recall**	**F1-Score**
**0**	**Visualization (0)**	**1.000**	**0.786**	**0.880**
	Workload (1)	0.824	1.000	0.903
	Macro average	0.912	0.893	**0.892**
1	Visualization (0)	0.000	0.000	0.000
	Workload (1)	0.364	0.571	0.444
	Macro average	0.182	0.286	**0.222**
2	Visualization (0)	0.500	1.000	0.667
	Workload (1)	0.000	0.000	0.000
	Macro average	0.250	0.500	**0.333**
3	Visualization (0)	0.933	1.000	0.966
	Workload (1)	1.000	0.929	0.963
	Macro average	0.967	0.964	**0.964**
4	Visualization (0)	0.481	0.929	0.634
	Workload (1)	0.000	0.000	0.000
	Macro average	0.241	0.464	**0.317**
5	Visualization (0)	0.500	0.929	0.650
	Workload (1)	0.500	0.071	0.125
	Macro average	0.500	0.500	**0.388**
6	Visualization (0)	0.000	0.000	0.000
	Workload (1)	0.417	0.714	0.526
	Macro average	0.208	0.357	**0.263**
7	Visualization (0)	0.733	0.786	0.759
	Workload (1)	0.769	0.714	0.741
	Macro average	0.751	0.750	**0.750**
Participant macro				**0.516** **±0.282**

Despite strong results for some participants, these data suggest that our initial model paradigm does not sufficiently capture patterns within the data suitable for realtime classification. Bold values indicate macro average F1-score per-participant.

### 3.2 LOO-CV results

**Figure 7:**
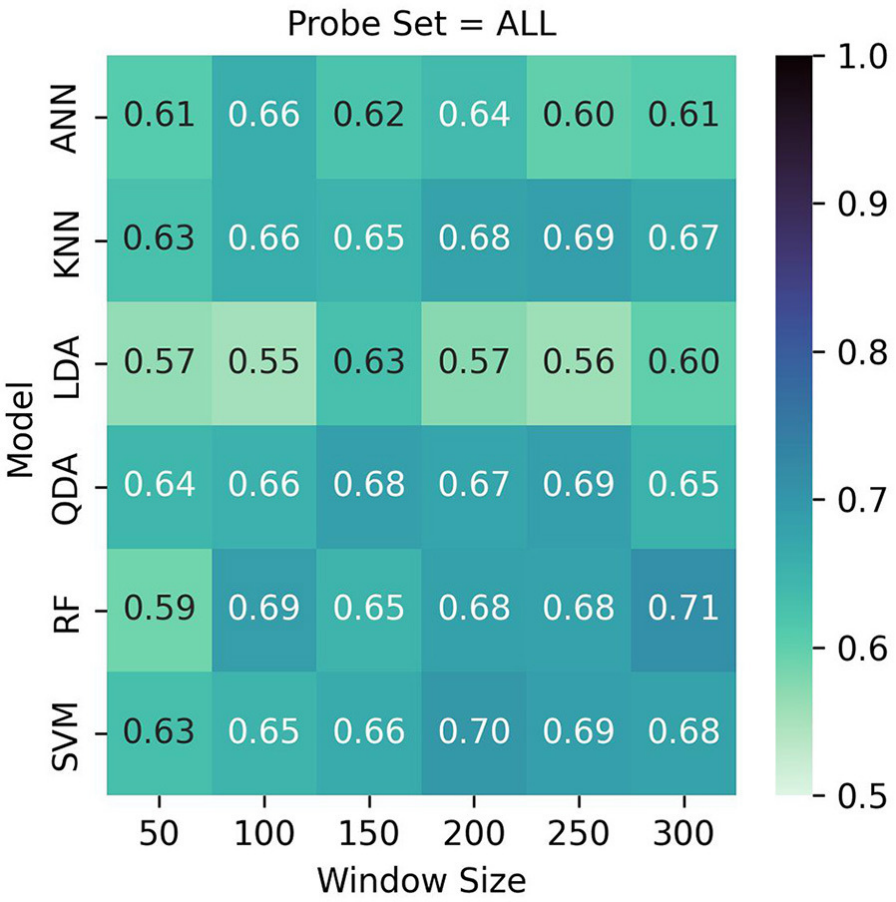
LOO cross validation results. Test set scores are produced based on the best model per-participant after inner hyperparameter optimization. Although Random Forest with a window size of 300 performed best with 71%, it showed similar results across multiple window sizes. QDA, KNN, and SVM likewise performed well overall.

**Figure 8:**
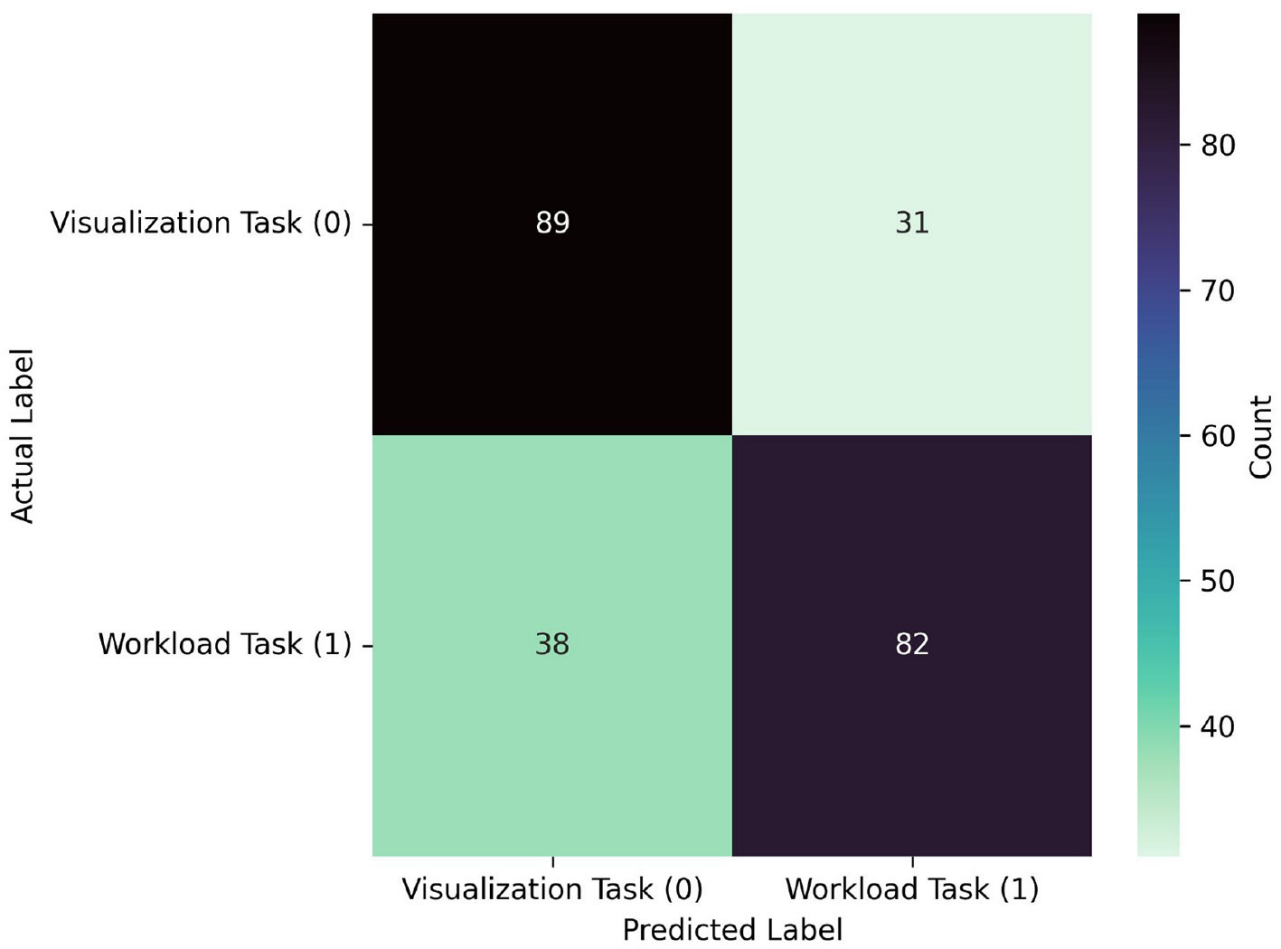
Confusion matrix for the best performing model from the LOO-CVV using all probes: RF, which achieved 0.710 at a window size of 300. The model predicted visualization and workload similarly well, correctly predicting 89 visualization samples, and 82 workload samples. The matrix also reveals relatively balanced misclassification patterns, with 31 visualization tasks misclassified as workload and 38 workload tasks misclassified as visualization.

See Table 2 and Figures 7, 8 for LOO-CV results. The RF classifier demonstrated the best performance overall per-window of 0.710 in the largest window size of 300, and achieved a mean F1-score of 0.667 across all window sizes. The model that produced this performance included a best overall F1 classification for the visualization task of 0.721, and similarly strong classification for the workload task of 0.704. However, SVM, KNN, and QDA all showed comparable overall effectiveness, with mean F1-scores of (0.669, 0.663, 0.665), respectively, across window sizes. The SVM classifier exhibited its best performance of 0.700 with a 200-sample window, KNN and QDA both performed best at the 250 sample window, each with top scores of 0.690. The ANN and LDA classifiers demonstrated the lowest overall effectiveness, with mean F1-scores of 0.623 and 0.580, respectively. While the ANN showed occasionally stronger performance with a maximum score of 0.660 at window size 100, LDA consistently underperformed compared to other methods, showing a maximum score of 0.630 at window size 100. Across models we observe a slight trend of better performance in visualization task detection compared to workload classification. Additionally, although most models showed improved performance with larger window sizes, this relationship is not strictly monotonic; further, the classification accuracies were similar enough such that a trade-off of slight accuracy for faster classification may be preferred in realtime contexts.

**Table 2:** Performance comparison across six machine learning models over varying window sizes in LOO-CV using all source data, showing F1-scores per-class for visualization and workload.

**Model**	**Metric**	**Window size**						**Mean**
		**50**	**100**	**150**	**200**	**250**	**300**	
RF	Visualization (0)	0.618	0.702	0.684	0.708	0.695	0.721	0.688
	Workload (1)	0.560	0.677	0.616	0.657	0.664	0.704	0.646
	Average	0.590	0.690	0.650	0.680	0.680	0.710	**0.667**
KNN	Visualization (0)	0.632	0.665	0.669	0.690	0.704	0.680	0.673
	Workload (1)	0.622	0.653	0.632	0.668	0.669	0.652	0.649
	Average	0.630	0.660	0.650	0.680	0.690	0.670	**0.663**
SVM	Visualization (0)	0.659	0.663	0.669	0.707	0.694	0.691	0.681
	Workload (1)	0.601	0.638	0.660	0.688	0.681	0.667	0.656
	Average	0.630	0.650	0.665	0.700	0.690	0.680	**0.669**
QDA	Visualization (0)	0.639	0.658	0.701	0.685	0.698	0.664	0.674
	Workload (1)	0.648	0.654	0.668	0.663	0.676	0.644	0.659
	Average	0.640	0.660	0.680	0.670	0.690	0.650	**0.665**
LDA	Visualization (0)	0.592	0.570	0.636	0.584	0.581	0.619	0.597
	Workload (1)	0.539	0.537	0.622	0.561	0.535	0.579	0.562
	Average	0.570	0.550	0.630	0.570	0.560	0.600	**0.580**
ANN	Visualization (0)	0.632	0.691	0.663	0.670	0.623	0.618	0.650
	Workload (1)	0.584	0.626	0.582	0.606	0.576	0.598	0.595
	Average	0.610	0.660	0.620	0.640	0.600	0.610	**0.623**

Results indicate promising performance across multiple models, including RF, KNN, SVM, and QDA. LDA, and ANN showed the least effectiveness in classification. Bold values indicate macro average F1-score across window sizes per-model.

### 3.3 Brain-network dependent classification

**Figure 9:**
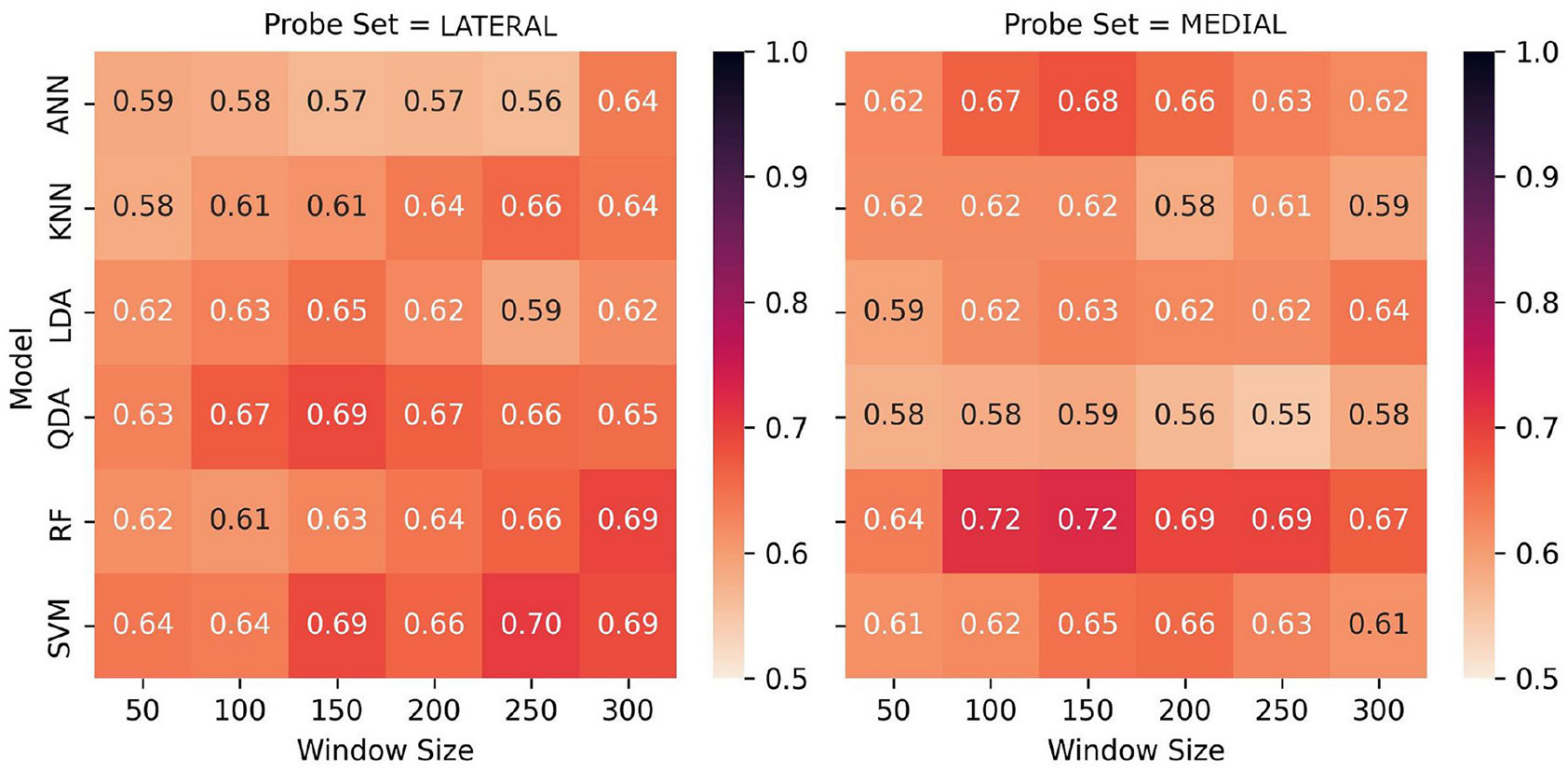
Performance comparison of models across window sizes using lateral and medial source sets, showing F1-scores for task classification. The heatmaps reveal distinctions in performance across probes for some models, with particular increases in performance for RF at lower window sizes for the medial probes, whereas SVM performed notably better using the lateral probes' data at higher window sizes.

**Figure 10:**
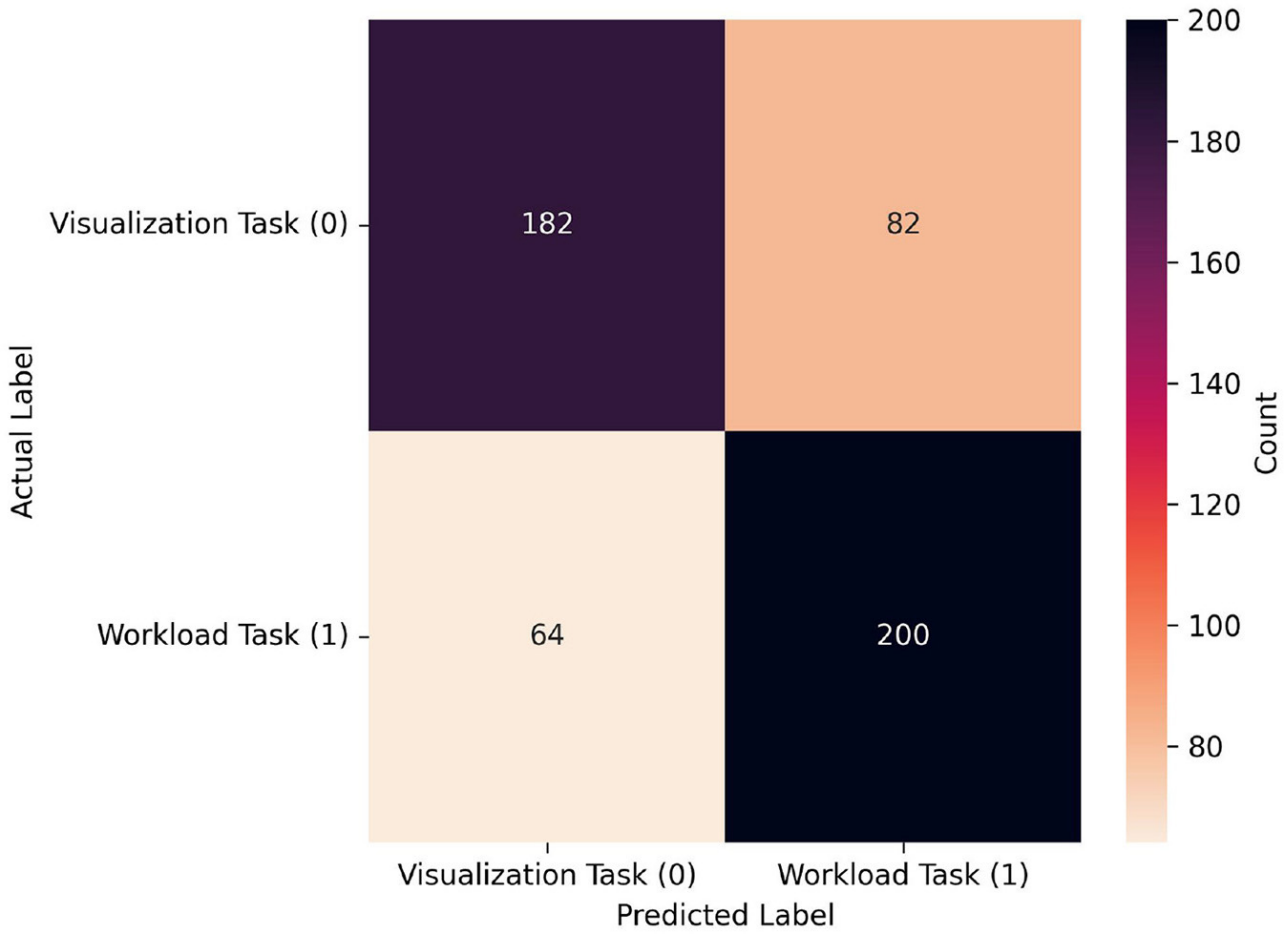
Confusion matrix for the best performing model from the LOO-CVV data when using limited source sets: RF, which achieved 0.723 at a window size of 150 when using data only from the medial source locations. As seen in the confusion matrix for the all-source data, proportions of classifications of both visualization and workload were similar, however in this case the workload task was correctly classified more often (200) than the visualization task (182), and workload was more often incorrectly classified (82) than visualization (64). Note that, given that the window size is half of that in Figure 8, the sample size is approximately double. In fact it is slightly larger, given that one extra sample could be gathered per-trial with this smaller window size.

Comparative analysis of lateral and medial source sets are presented in Table 3 and visualized in Figures 9, 10. Several notable patterns are visible across both source locations and temporal windows in the data. The lateral source data showed particular sensitivity to window size selection, with the best performing models demonstrating improved performance at larger temporal windows: SVM had the strongest overall performance, with a maximum F1-score of 0.705 at a 250-sample window, and an average F1-score of 0.671 across window sizes. This performance was closely matched by RF with a score of 0.695 at 300 samples, and a slightly lower overall performance across windows of 0.642. QDA presented an interesting departure from the trend of higher classification accuracies with larger window sizes, demonstrating its best performance of 0.690 at window size of 150—while its overall accuracy of 0.662 was better than RF, its maximum performance was not as good. As with the full-source data, LDA and ANN underperformed by comparison to the other models, with accuracies of 0.622 and 0.648, respectively.

**Table 3:** Model performance when trained on subsets of only the relatively medial or lateral sources across varying window sizes.

**Model**	**Source set**	**Window size**						**Mean**
		**50**	**100**	**150**	**200**	**250**	**300**	
RF	LATERAL	0.616	0.607	0.626	0.643	0.664	0.695	0.642
	MEDIAL	0.635	0.717	0.723	0.693	0.694	0.670	0.689
KNN	LATERAL	0.583	0.605	0.612	0.641	0.659	0.642	0.624
	MEDIAL	0.621	0.620	0.621	0.583	0.614	0.592	0.609
SVM	LATERAL	0.641	0.636	0.691	0.664	0.705	0.687	0.671
	MEDIAL	0.613	0.622	0.650	0.656	0.628	0.612	0.630
QDA	LATERAL	0.630	0.673	0.690	0.667	0.656	0.654	0.662
	MEDIAL	0.578	0.582	0.586	0.565	0.547	0.584	0.574
LDA	LATERAL	0.616	0.632	0.651	0.624	0.589	0.621	0.622
	MEDIAL	0.592	0.619	0.630	0.622	0.621	0.641	0.621
ANN	LATERAL	0.586	0.580	0.568	0.572	0.563	0.637	0.584
	MEDIAL	0.625	0.668	0.682	0.656	0.635	0.622	0.648

Results from the medial sources revealed different patterns from the lateral. At lower window sizes of 100 and 150, RF demonstrated the best performance overall of 0.717 and 0.723, besting the best performance from all models trained across both datasets. RF also demonstrated the highest classification accuracy across window sizes, with an average of 0.689. Performance disparity between source sets was largest for QDA, which maintained 0.63-0.69 with the lateral sources but decreased to 0.55–0.59 with the medial sources. Although LDA showed the most consistent performance across both source sets, maintaining F1-scores between 0.59-0.65 regardless of location, the overall performance of this classifier was lower than other methods. The asymmetry in classifications among the better-performing models suggests that there are significant differences in the underlying data distributions between source locations as related to task-based activation.

We believe that our findings suggest an opportunity for enhanced performance through meta-classification approaches. Specifically, the data shows that each source location exhibits unique strengths in capturing task-related neural states: of most notable distinction, the medial source set demonstrates exceptional performance with RF, reaching F1-scores of 0.72 at moderate window sizes, while the lateral source set shows particular strength with SVM, achieving F1-scores of 0.70 with larger windows. This performance asymmetry, as hypothesized by Hincks et al. (2017), supports the importance of considering network interactions, but suggests a novel approach to leveraging these interactions. Rather than rely on simultaneous bilateral measurements for direct network comparison, our results indicate that independent classification streams from each source location and temporal samples could be combined through a meta-classifier architecture. This approach would capitalize on the complementary strengths we observed from different models based on probes: RF with the medial probes, and SVM and QDA with the lateral probes. Further, the distinct temporal window preferences between source sets (medial probes performing optimally at 100–150 samples, lateral probes at 250–300 samples) could further be supported by a meta-classification approach over multiple window lengths.

## 4 Conclusion

We developed a real-time implicit fNIRS-BCI study based on the vision of the leveraging of brain networks toward a next-generation memory prosthesis interface using fNIRS. Although our online real-time classification was not superb, offline simulations of real-time classification which leveraged a larger dataset, longer window times, and a wider feature set show great promise for future tasks leveraging brain-network based tasks for applied BCI which are suitable for cross-participant classification across multiple classifiers. Further, our results suggest a promising direction for future system development: implementing parallel classification streams that independently process signals from each source location, and potentially at different window lengths, then combining these predictions through a higher-level meta-classifier. Such an architecture could maintain the benefits of bilateral monitoring while accounting for distinct information patterns to be captured at each location. We believe that future extension of such interfaces with broader access to the brain will be able to provide wider and more comprehensive interfaces based on more complex sets of human state information. While real-time performance requires further optimization, this study represents a significant advancement in brain network-based BCIs, potentially leading to more intuitive interfaces that facilitate information access based on mental states.

## Data Availability

The raw data supporting the conclusions of this article will be made available by the authors, without undue reservation.
